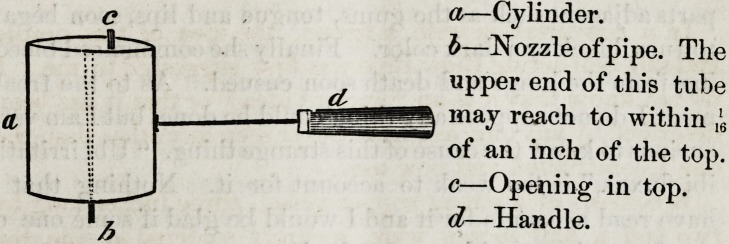# Notes from Dental Practice

**Published:** 1869-02

**Authors:** William T. Russell


### ARTICLE IV.

### *Notes from Dental Practice.*

---

### *A Self-acting Blow Pipe.*

By William T. Russell, D.D.S.

In a conversation with a friend, the idea of this simple and safe Self-acting Blow Pipe was first suggested to me. I do not know that it is an " ancient idea " or that it has been described by any one, as I have never seen any other than the one I constructed and now use.

It consists of a strong copper vessel, three inches long by two and a half inches wide, securely soldered at side, top and bottom. From the under side of the top, through the axis of the cylinder, and perforating the centre of the bottom projects a tapering tube, which at its top has a diameter of $\frac{1}{4}$ of an inch, and at the lower end $\frac{1}{8}$ of an inch. In order to give a proper opening to this nozzle of the pipe, I soldered a thin plate over the end, and then with a hair like drill bored a *small* and even hole. This tube is soldered firmly in its place. Through the top is an $\frac{1}{8}$ inch opening to be stopped with soft wood. The handle is made of sufficiently strong wire, one end of which is inserted into wood, the other is soldered to the middle of the cylinder. The manipulation is very simple. Into the small opening on the top, pour from one-half to one ounce of alcohol and stop with soft wood. I hold the cylinder over the flame till the alcohol is vaporized, then by the same manipulations as with the mouth Blow Pipe you may have a blast—broad and full, or intense and pointed, as may be required. Should the alcohol vaporize too rapidly, and so generate a great " head of steam " the pressure on the conical plug of soft wood expells it, and the danger ends, But there is no necessity to have so much pressure, for with a sufficiently small opening (1) in the nozzle (2)—(which should be hair-like) the blast is ample to direct any ordinary soldering flame.

I have used this little hand Blow Pipe for melting gold and silver, for heating up dentures imbedded in plaster and sand, and on several occasions have completed the soldering of the entire piece with it.

The subjoined illustration of the instrument will make its structure and use easily comprehended.

*a*—Cylinder.
*b*—Nozzle of pipe. The upper end of this tube may reach to within $\frac{1}{16}$ of an inch of the top.
*c*—Opening in top.
*d*—Handle.

### *Fungous Growth of Nerve. Death from the Extraction of a Tooth.*

By C. W. Westmoreland, D.D.S.

I would like to report to your Journal, through you, several cases which have come under my observation recently.

The first of the cases to which I refer is that of a first superior molar. This and the corresponding molar on the opposite side were so badly decayed that it was necessary for me to destroy the nerves. I applied a nerve paste made of arsenic, creosote and morphia, according to a recipe learned at college, and had fine success with one, which I filled. The other appeared obstinate and did not yield until the second or third application. After the last application, it was necessary for my patient to be absent for about a month. When he returned, the cavity was filled with flesh, which I thought was *gum*, I cut it away and told him to return in a day or two and I would commence the treatment preparatory to filling. But he was again called away, and returned after some time, when I again found the cavity filled with flesh. After cutting it away, and probing the canals, I am *satisfied* that this growth is from the nerve canals. The questions I wish to ask are as to the causes and treatment. The patient will not consent to have the tooth extracted. I have had excellent success in the treatment of fangs, *never have failed* in a single case.

The 2nd case is as follows:

An old lady about 75 or 80 years of age was very much annoyed by a lower front tooth which was very loose. She had some one to pull it (with a string I think). It began to bleed very freely and was arrested by some styptic, but the parts adjacent such as the gums, tongue and lips, soon began to turn black or a dark color. Finally she commenced bleeding from the lungs and death soon ensued. As to the treatment I do not suppose any thing could be done, but I am very curious to know the cause of this strange thing. "Ubi irritatio ibi fluxus," is too weak to account for it. Nothing that I have read accounts for it and I would be glad if some one of the fraternity *would* account for it.

In regard to the first case to which Dr. Westmoreland refers—fungous growth of gum—the general rule is to extract all teeth affected with these vascular tumors, as they are certain to be reproduced in nearly all cases where attempts are made to destroy them by extirpation. In the case of a 1st bicuspid with a single root, which came under our care, the patient objected so earnestly to the extraction of the tooth that we determined to use all means in our power to preserve it, and in this case succeeded by the following treatment, although the same treatment attempted afterwards in the case of a superior molar failed: After extirpating the vascular tumor and finding it reproduced, it was again extirpated, this time more carefully than the first, and as far as the foramen in the end of the root. An application of nitrate of silver was then made by introducing a small portion in a solid form into the pulp canal, and carrying it, by means of a nerve instrument, as far as the foramen, and the crown cavity filled with a temporary filling of cotton saturated with sandarach varnish. An engagement was made for the following day, at which time the pulp canal was filled with gold as high up as it is usual to carry fillings of this kind. Prior to introducing the gold a small pellet of cotton saturated with carbolic acid was introduced and the gold inserted over this. For several days there was an uneasy feeling in the tooth which did not, however, amount to actual pain, and which passed off at the end of this time. For several months afterwards the tooth remained quiet, and as we lost sight of the patient then and have not seen him since, we feel justified in concluding that the treatment as described was successful.

As regards the second case of Dr. Westmoreland—death from hæmorrhage following extraction of a tooth—it was clearly one of a hæmorrhagic diathesis. The blood must have been in a depraved condition, what is known as "spanæmia," a condition in which the blood is thin and poor, wanting in fibrin. There was also a loss of the recuperative power necessary to bring about the restoration and healing of the parts. Cases of the same fatal nature have before been recorded, where all known means failed to arrest the flow of blood, and where almost the entire surface of the mucous membrane of the mouth poured forth such a continuous stream as soon to produce fatal exhaustion. The age of the patient in the case referred to by Dr. W. together with a depraved condition of the system, evidently caused the application of the styptic to be of no avail. The dark color of the parts was caused by the loss of vitality, resulting in mortification. If a solution of the persulphate of iron or powdered subsulphate, when properly applied fail to arrest hæmorrhage the case is indeed a desperate one. We do not think, however, that these agents or any other would have succeeded in the case referred to. (Ed).

## Figures and Tables

**Figure f1:**